# Supplementary material for: Walk Longer! Using Wearable Inertial Sensors to Uncover Which Gait Aspects Should Be Treated to Increase Walking Endurance in People with Multiple Sclerosis
Source: Sensors (Basel). 2024 Nov 14;24(22):7284. doi: 10.3390/s24227284 (PMC11598194; doi:10.3390/s24227284)
Supplement: Supplementary file 1 [file sensors-24-07284-s001.zip › sensors-3278870-supplementary.pdf]

Supplementary Material

Table S1. Results of the multivariate linear regression analysis with the Six-Minute Walk Test (6MWT) as the dependent variable (whole sample of PwMS, *N* = *56*). The model is adjusted for age, sex, and use of walking aids.

**Adjusted R^2**	***p*-Value ($F_{8,47}$)**	**Independent Variable (Domain)**	**b (SE)**	**β (SE)**	***p*-Value (t_{47})**
0.91	< 0.001 (73.60)	Double Support * (Rhythm & Pace)	-4.69 (0.58)	-0.43 (0.05)	< 0.001 (-8.08)
		Stride Regularity Mod. * (Regularity/Variability)	185.73 (50.72)	0.26 (0.07)	< 0.001 (3.66)
		iHR AP (Gait Symmetry)	0.89 (0.68)	0.07 (0.06)	0.197 (1.31)
		nRMS ML * (Trunk Sway)	-116.13 (35.20)	-0.18 (0.05)	0.002 (-3.30)
		$sLyE_{step}$ AP * (Gait Instability)	-159.22 (37.47)	-0.20 (0.05)	< 0.001 (-4.25)

PwMS: people with MS; iHR: improved Harmonic Ratio; $sLyE_{step}$: short-term Lyapunov exponent over one step; nRMS: normalized root mean square of trunk acceleration; Mod.: trunk acceleration modulus; AP: antero-posterior; ML: medio-lateral; SE: standard error. * *p*-Value < 0.05.

Table S2. Results of the multivariate linear regression analysis with the Six-Minute Walk Test (6MWT) as the dependent variable (subsample of $PwMS_{mFR}$, *N* = *23*). The model is adjusted for age, sex, and use of walking aids.

Adjusted R^2	*p*-Value ($F_{8,14}$)	Independent Variable (Domain)	b (SE)	β (SE)	*p*-Value (t_{14})
0.87	< 0.001 (20.09)	Double Support * (Rhythm & Pace)	-5.81 (1.67)	-0.43 (0.12)	0.004 (-3.48)
		Stride Regularity Mod. * (Regularity/Variability)	385.68 (87.14)	0.53 (0.12)	< 0.001 (4.43)
		iHR AP (Gait Symmetry)	-1.07 (0.98)	-0.11 (0.10)	0.293 (-1.09)
		nRMS ML (Trunk Sway)	-69.60 (50.50)	-0.19 (0.14)	0.190 (-1.38)
		$sLyE_{step}$ AP † (Gait Instability)	-133.19 (71.73)	-0.20 (0.11)	0.084 (-1.86)

$PwMS_{mFR}$: people with MS at minimal fall risk; iHR: improved Harmonic Ratio; $sLyE_{step}$: short-term Lyapunov exponent over one step; nRMS: normalized root mean square of trunk acceleration; Mod.: trunk acceleration modulus; AP: antero-posterior; ML: medio-lateral; SE: standard error. * *p*-Value < 0.05; † *p*-Value < 0.1.

Table S3. Results of the multivariate linear regression analysis with the Six-Minute Walk Test (6MWT) as the dependent variable (subsample of $PwMS_{FR}$, *N* = *33*). The model is adjusted for age, sex, and use of walking aids.

Adjusted R^2	*p*-Value ($F_{8,24}$)	Independent Variable (Domain)	b (SE)	β (SE)	*p*-Value (t_{24})
0.78	< 0.001 (14.88)	Double Support * (Rhythm & Pace)	-4.21 (0.65)	-0.65 (0.10)	< 0.001 (-6.51)
		Stride Regularity Mod. (Regularity/Variability)	106.63 (60.72)	0.20 (0.12)	0.100 (1.71)
		iHR AP (Gait Symmetry)	0.33 (0.87)	0.03 (0.09)	0.703 (0.39)
		nRMS ML * (Trunk Sway)	-121.74 (46.09)	-0.27 (0.10)	0.014 (-2.64)
		$sLyE_{step}$ AP * (Gait Instability)	-103.90 (46.30)	-0.23 (0.10)	0.034 (-2.24)

$PwMS_{FR}$: people with MS at moderate-high fall risk; iHR: improved Harmonic Ratio; $sLyE_{step}$: short-term Lyapunov exponent over one step; nRMS: normalized root mean square of trunk acceleration; Mod.: trunk acceleration modulus; AP: antero-posterior; ML: medio-lateral; SE: standard error. * *p*-Value < 0.05.

Table S4. Digital metrics descriptive of gait in HS and PwMS with comparable walking speed.

Metric	HS *(N = 10)*	PwMS *(N = 20)*	***p*-Value**
Straight-line Gait Speed [m/s]	1.6 (1.5; 1.8)	1.6 (1.5; 1.7)	0.202
Double Support [%stride duration]	12.2 (8.4; 15.0)	11.5 (8.6; 15.0)	0.948
Stride Regularity Mod. [unitless]	0.93 (0.89; 0.94)	0.89 (0.84; 0.92)	**0.044**
iHR AP [unitless]	89.8 (84.7; 93.7)	78.7 (75.9; 86.0)	**< 0.001**
nRMS ML [unitless]	0.47 (0.41; 0.55)	0.64 (0.48; 0.82)	**0.044**
$sLyE_{step}$ AP [unitless]	0.57 (0.55; 0.64)	0.65 (0.60; 0.74)	**0.031**

Values are median (25th;75th percentiles). HS: healthy subjects; PwMS: people with MS; iHR: improved Harmonic Ratio; $sLyE_{step}$: short-term Lyapunov exponent over one step; nRMS: normalized root mean square of trunk acceleration; Mod.: trunk acceleration modulus; AP: antero-posterior; ML: medio-lateral. *p*-Value: result of the Mann-Whitney U test (HS vs. PwMS). Statistically significant values ($p < 0.05$) are reported in bold.

Table S5. Number (percentage) of people with MS with different severity levels (EDSS) showing abnormal values of instrumented metrics.

**Domain**<br>Metric	**Cut-off Value**	**Mild**<br>*(N = 6)*	**Moderate**<br>*(N = 27)*	**Severe**<br>*(N = 23)*
**Rhythm & Pace**				
Double Support	> 15.8<br>%stride duration	0 (0%)	12 (44%)	20 (87%)
**Regularity/Variability**				
Stride Regularity Mod.	< 0.87<br>unitless	1 (17%)	17 (63%)	22 (96%)
**Gait Symmetry**				
iHR AP	< 80.2<br>unitless	3 (50%)	19 (70%)	22 (96%)
**Trunk Sway**				
nRMS ML	> 0.70<br>unitless	1 (17%)	15 (56%)	16 (70%)
**Gait Instability**				
$sLyE_{step}$ AP	> 0.67<br>unitless	3 (50%)	16 (59%)	15 (65%)

EDSS: Expanded Disability Status Scale; Mild: people with mild MS (EDSS: 0-2.5); Moderate: people with moderate MS (EDSS: 3-5.5); Severe: people with severe MS (EDSS: 6-6.5); iHR: improved Harmonic Ratio; $sLyE_{step}$: short-term Lyapunov exponent over one step; nRMS: normalized root mean square of trunk acceleration; Mod.: trunk acceleration modulus; AP: antero-posterior; ML: medio-lateral.
